# Exploring Mechanisms for Effective Technology-Enhanced Simulation-based Education in Wilderness Medicine: A Systematic Review

**DOI:** 10.7759/cureus.412

**Published:** 2015-12-17

**Authors:** Ralph MacKinnon, Deborah Aitken, Christopher Humphries

**Affiliations:** 1 Department of Paediatric Anaesthesia & North West and North Wales Paediatric Transport Service, Royal Manchester Children's Hospital, UK; 2 Research and Innovation, Royal Manchester Children's Hospital, UK; 3 Royal Preston Hospital, Preston, UK

**Keywords:** medical simulation, systematic review, wilderness medicine, technology, education, learner outcomes

## Abstract

Background: Technology-enhanced simulation is well-established in healthcare teaching curricula, including those regarding wilderness medicine. Compellingly, the evidence base for the value of this educational modality to improve learner competencies and patient outcomes are increasing.

Aims: The aim was to systematically review the characteristics of technology-enhanced simulation presented in the wilderness medicine literature to date. Then, the secondary aim was to explore how this technology has been used and if the use of this technology has been associated with improved learner or patient outcomes.

Methods: EMBASE and MEDLINE were systematically searched from 1946 to 2014, for articles on the provision of technology-enhanced simulation to teach wilderness medicine. Working independently, the team evaluated the information on the criteria of learners, setting, instructional design, content, and outcomes.

Results: From a pool of 37 articles, 11 publications were eligible for systematic review. The majority of learners in the included publications were medical students, settings included both indoors and outdoors, and the main focus clinical content was initial trauma management with some including leadership skills. The most prevalent instructional design components were clinical variation and cognitive interactivity, with learner satisfaction as the main outcome.

Conclusions: The results confirm that the current provision of wilderness medicine utilizing technology-enhanced simulation is aligned with instructional design characteristics that have been used to achieve effective learning. Future research should aim to demonstrate the translation of learning into the clinical field to produce improved learner outcomes and create improved patient outcomes.

## Introduction

Both professionals and patients are becoming increasingly aware of the potential high-risk adverse events that can occur in medicine [1]. Likewise, a growing body of evidence supports the approach that technology-enhanced simulation can identify deficits and make improvements to learner competency in healthcare settings [2-4]. Therefore, technology-enhanced simulation can be used as a way of decreasing this risk through the development of clinical knowledge, skills, behaviors, and attitudes within a risk-free environment. This development occurs by the learner interacting with the technology-enhanced simulation tool or device to mimic an aspect of clinical care for the purpose of teaching or assessment [5]. This simulation technology-enhanced education method encompasses a spectrum of educational modalities from computer-based gaming and virtual learning to body part task trainers (anatomical representation of body parts), highly realistic electronic manikins (termed human patient simulators), and simulated patients.

As learners are not practicing on patients, simulation allows acquisition and development of these medical competencies without the risk of endangering themselves, their reputation, or the general public. Errors that occur while practicing through simulation can be a resource for learning by raising awareness of deficits in performance. This provides educational facilitators with the opportunity to correct errors and provide feedback until mastery is achieved, preventing a patient being harmed through suboptimal care [6-7]. This feedback on observed simulation performance is often referred to as a facilitated debriefing and fits within Rudolph, et al.’s suggested four-step model of simulation-based learning: identifying performance gaps related to predetermined objectives, providing feedback describing the gap, investigating the basis for the gap, and helping to close the gap through discussion and targeted instructions [8].

A recent meta-analysis of simulation for health professions education reported better learning outcomes in knowledge, procedural skills, and behaviors with technology-enhanced simulation education compared to traditional educational practice [9]. The realizable value of technology-enhanced simulation has been demonstrated extensively in clinical skills improvements (airway management, CPR training), reduction in surgical mortality, reduction in annual obstetrical malpractice premiums, reduction in bloodstream infections, and the recognition of latent safety threats in healthcare systems [3-4, 10-14]. Therefore, simulation education in health care is both an effective and a valid training tool that leads directly to better patient outcomes.

The use of simulation to teach patient assessment is well established in wilderness medicine [15]. Militaries around the world utilize human patient simulator technology in their training, and as a consequence, many simulators have been developed to be robust [16]. Additionally, recent technological developments, including robotics, pneumatics, and Wi-Fi, have resulted in human patient simulators without large cables linking to generators or monitors. Therefore, all the requisite components can fit in an easily portable container dependent on the size of the patient chosen. Consequently, such robust and technologically advanced human patient simulators can be used in training by wilderness medicine teams whilst being controlled by a handheld controller out of sight of participants.

Current evidence supports following the 11 instructional design components as best practice in technology-enhanced simulation-based training: clinical variation, cognitive interactivity, curricular integration, distributed practice, feedback, group versus independent practice, individualized learning, mastery learning, multiple learning strategies, learning over a long time (distributive practice), range of task difficulty, and repetitive practice [7, 9]. It is recognized that the value of one technology-enhanced simulation approach compared to another is context-specific, so the value of a given simulated approach may be greater or lesser depending on the educational context and learning objectives. Following a series of systematic reviews provides an insight into the instructional design components and the educational mechanics that define effective simulation-based training [7, 9]. However, at present, the realizable value of simulation in wilderness medicine relative to other traditional models of teaching is unknown. Likewise, the relative merits of different technology-enhanced simulation approaches in wilderness medicine simulation training is unknown.

## Materials and methods

### Aim and objectives

In this review, the aim was to explore the mechanisms of effective simulation-based education published to date in the wilderness medicine literature with a view to providing insight for the future development of this field. To accomplish this, the following objective questions were used:

What characteristics of technology-enhanced simulation have been presented in the wilderness medicine literature to date?

How has this technology-enhanced simulation been used in the wilderness medicine literature to date?

Has the use of technology-enhanced simulation in wilderness medicine been associated with improved learner outcomes and improved patient outcomes?

### Inclusion and exclusion criteria

Articles included were published in English, described the utilization of any technology-enhanced simulation modality to teach health professionals any aspect of wilderness medicine at any stage of training or practice, and used a modification of the Kirkpatrick outcomes of satisfaction, knowledge or attitudes, skills and behaviors (in practice), or effects on patients [17-18].

Articles excluded were not in English, did not mention any aspect of technology-enhanced simulation (including standardized or simulated patients), or were only available in abstract, not full-text format.

A search for articles on simulation training in wilderness medicine for the years 1946 to 2014 was conducted using the OVID Medline and Embase databases as well as other non-indexed citation-based databases. Terms used in the search were: wilderness, education, simulation, wilderness medicine, manikins, medical education, computer simulation, patient simulation, and standard patient. The reference sections of those articles were also reviewed for any articles not indexed in Medline/PubMed. The concluding search was conducted in August 2014.

Working independently and, therefore, in replicate, the authors reviewed the titles and abstracts yielded by the search strategy. If insufficient information was present in the abstract, the full-text article was reviewed for inclusion. In the case of any disagreements, the full-text articles were reviewed and consensus achieved.

### Definitions

In order to characterize how the simulation technology has been utilized in each study, the instructional design characteristics defined in previous reviews on health professions education have been used [19-20]. Wilderness medicine health professionals were defined as students, postgraduate trainees (residents, specialist trainees, or fellows), or practitioners in a profession directly related to wilderness medicine. The technology-enhanced simulation was defined as an educational tool or device with which the learner interacts to mimic an aspect of clinical care for the purpose of teaching or assessment [9]. Wilderness medicine was defined as healthcare delivered in remote settings without rapid access to additional resources.

### Measures and coding

A data abstraction form was created to capture the following information from each article selected:

Overview of article characteristics: Training level of healthcare learners participating, setting/location of educational provision, clinical topics described, and design of article.

Instructional design features: Clinical variation, cognitive interactivity, curricular integration, distributed practice, feedback, group versus independent practice, individualized learning, mastery learning, multiple learning strategies, the range of task difficulty, and repetitive practice.

Outcome measures: Kirkpatrick Level 1 - reaction to learning experience; Kirkpatrick Level 2a - modification of attitudes and perceptions; Kirkpatrick Level 2b - acquisition of knowledge and skills; Kirkpatrick Level 2c - retention of knowledge and skills; Kirkpatrick Level 3 - behavioral change; Kirkpatrick Level 4a - change in organizational practice; and Kirkpatrick Level 4b - benefits to patients/clients, families, and communities [17-18].

Articles were catalogued by characteristics, features, and Kirkpatrick levels according to the article description. Some articles described more than one characteristic, feature, or Kirkpatrick level; in these cases, all were recorded and included in the analysis. From this qualitative data assessment, the emergence of current themes and deficits were explored.

## Results

**Figure 1:**
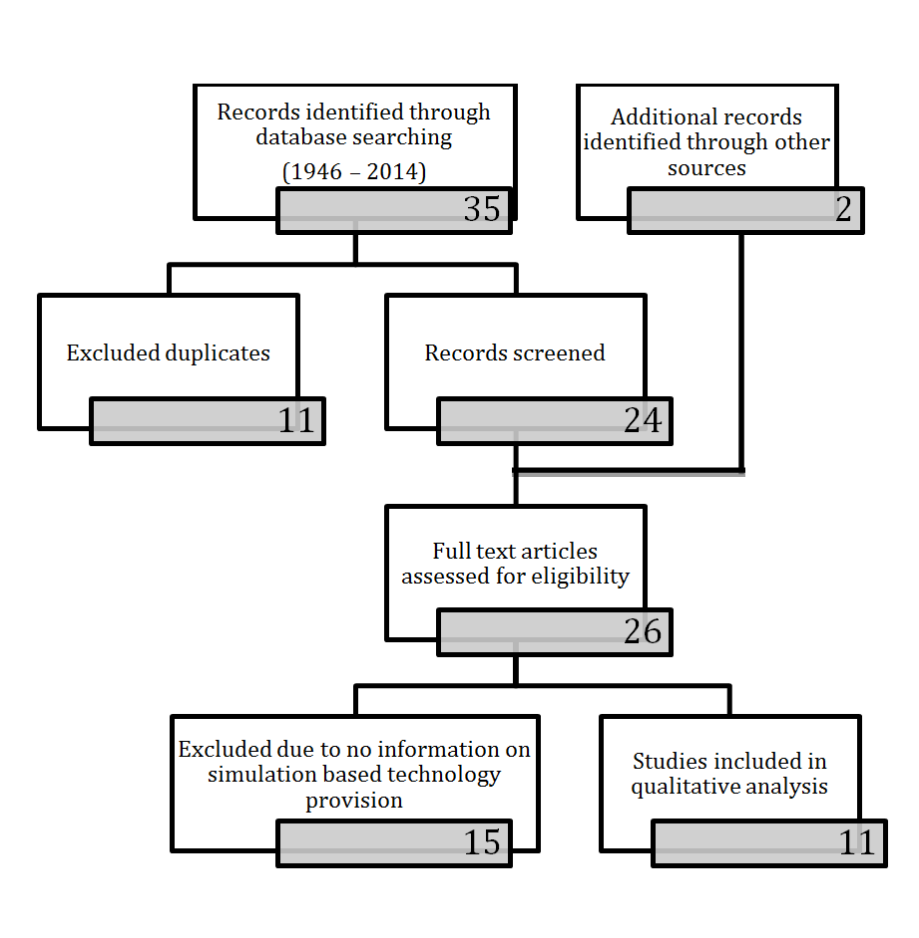
Study Flow Diagram

The search strategy revealed 35 articles and a further two articles from the review of references. After removal of duplicates, 26 articles were reviewed and 15 excluded as no information on the characteristic of technology-enhanced simulation could be discerned. Therefore, 11 articles were ultimately included in this review (Figure 1).

### Overview of article characteristics

Table 1 summarises the article characteristics found in this review.

**Table 1:** Overview of Included Articles

Date	Authors	Country	Participants	Location	Clinical Topics	Design
2014	Saxon, et al. [21]	USA	Medical students	Large arboretum	Rapid scene assessment, appropriate care, and stabilization. Emergency procedural skills.	Evaluation of course and description of low fidelity simulation models
2013	Lockwood, et al. [22]	Scotland, UK	Medical students	Campsite	First aid, expedition medicine, leadership, working outdoors, emergency care.	Description and evaluation of course
2011	Fielding [23]	USA	Medical students	Trailhead	Scene and patient assessment, limb and spinal immobilization, equipment adaptation, medical kit design	Description and evaluation of course
2011	Mazoyer, et al. [24]	England UK	Medical students	Countryside	Assessment and treatment of traumatic injuries, planning, and further management, communication, navigation and team-working	Description of course
2010	Lareau, et al. [30]	USA	Students – not stated medical or otherwise	Countryside	Emergency trauma care	Description of course, discussion of use of a high technology patient simulator in a wilderness environment
2010	Heiner, et al. [28]	USA	Emergency Medical Technicians	Emergency Department	Fracture detection by ultrasonography	Evaluation of a fracture simulation model
2009	Andrews, et al. [25]	England UK	Medical Students	Classroom and countryside	Casualty management in adverse situations and environments, teamwork	Description of course (participant perspective)
2004	Macias, et al. [26]	USA	Medical Students	Countryside and indoor simulations	Resuscitation, rescue, environmental medicine, flora and fauna, travel medicine	Description and evaluation of course
2000	Donelan [15]	USA	Students – not stated medical or otherwise	Outdoors and indoors	Simulation techniques, standardized simulation, feedback	Opinion article
2000	Vohra, et al. [29]	USA	Doctors, nurses, students, paramedics, national park medics, and wilderness enthusiasts	Emergency department, grand rounds, and conference venues	Assessment and management of venomous injuries	Description and evaluation of course
1997	Houghton [27]	USA	Medical students	Canyon	Head injury, patient transport, cardiopulmonary resuscitation	Description of course

Design of Article:

All articles used technology-enhanced simulation (including standardized/simulated patients) to teach wilderness medicine clinical skills (e.g., trauma care), non-technical skills (e.g., leadership), and adaptation to adverse environments.

Training Level of Healthcare Learners Participating:

The learners described were solely medical students in seven of the articles [21-27]. Two studies included emergency medical technicians or paramedics [28-29]. Finally, in two articles, the participants were described as students (but not specified as students of medicine) [15, 29]. In one article, doctors, nurses, students, paramedics, national park medics, and wilderness enthusiasts were included as participants [29].

Setting/Location of Educational Provision:

The majority of locations for teaching were outdoors [15, 21-27]. However, indoor simulation of external environments was also described [15, 25-26, 28-30]. External environments ranged from UK countryside to US trailheads, canyons, and campgrounds.

Clinical Topics Described:

The majority of clinical topics taught focused upon knowledge acquisition for the initial management of traumatically injured patients, including scene and patient assessment (primary and secondary survey) and stabilization. The development of procedural skills using simulation was described in detail in seven articles [21, 23, 25-28, 30]. The use of simulation to develop non-technical skills of leadership, communication, situational awareness (including planning ahead), and decision-making was evident in five articles [22, 24-27]. Ten of the articles reviewed provided a description of a wilderness medicine course [21-30]. Seven of those 10 articles provided an evaluation of the course/educational intervention [21-23, 25, 28-29]. One article provided an overview on realism techniques and insights into standardized/simulated patients and feedback [15]. Finally, four articles detailed novel innovations to teaching wilderness medicine [15, 21, 28, 30].

### Instructional design features

In Table 2 below, we show the instructional design features of the articles in this review.

**Table 2:** Instructional Design Characteristics of Articles Reviewed Characteristics modified from Cook, et al. 2013 [5]

Instructional Design Characteristics	Operational definition	Articles describing characteristics
Clinical variation	Variation in the clinical context, for example, multiple different patient scenarios.	Donelan [15] Saxon, et al. [21] Lockwood, et al. [22] Fielding [23] Mazoyer, et al. [24] Andrews, et al. [25] Macias, et al. [26] Heiner, et al. [28] Vohra, et al. [29] Lareau, et al. [30]
Cognitive interactivity	Training that promotes learners’ cognitive engagement using strategies, such as task variation and or intentional task sequencing multiple repetitions and feedback.	Donelan [15] Saxon, et al. [21] Lockwood, et al. [22] Fielding [23] Mazoyer, et al. [24] Andrews, et al. [25] Macias, et al. [26] Heiner, et al. [28] Vohra, et al. [29] Lareau, et al. [30]
Curricular integration	Incorporation of the simulation intervention as an integral part (required or formal element) of the curriculum or training program.	Macias, et al. [26]
Distributed practice	Training spread over a period of time, interventions that involved 41 days of simulation training.	
Feedback	Information on performance provided to the learner by the instructor, a peer, or a computer, either during or after the simulation activity.	Donelan [15] Saxon, et al. [21] Lockwood, et al. [22] Andrews, et al. [25] Macias, et al. [26] Houghton [27] Vohra, et al. [29] Lareau, et al. [30]
Group (versus independent) practice	Training activities involving two or more learners	Donelan [15] Saxon, et al. [21] Lockwood, et al. [22] Fielding [23] Mazoyer, et al. [24] Andrews, et al. [25] Macias, et al. [26] Houghton [27] Vohra, et al. [29] Lareau, et al. [30]
Individualized learning	Training responsive to individual learner needs (i.e. tailored or adapted depending on performance).	Saxon, et al. [21] Mazoyer, et al. [24] Andrews, et al. [25] Macias, et al. [26]
Mastery learning	Training model in which learners must attain a clearly defined standard of performance before qualifying or advancing to the next task.	
Multiple learning strategies	The number of different instructional strategies used to facilitate learning, such as patient case, worked example, discussion, feedback, intentional sequencing, or task variation.	Saxon, et al. [21] Lockwood, et al. [22] Fielding [23] Mazoyer, et al. [24] Andrews, et al. [25] Macias, et al. [26] Vohra, et al. [28] Lareau, et al. [30]
Range of task difficulty	Variation in the difficulty or complexity of the task (explicitly stated).	Donelan [15] Saxon, et al. [21] Mazoyer, et al. [24] Andrews, et al. [25] Macias, et al. [26]
Repetitive practice	The opportunity for more than one task performance.	Mazoyer, et al. [24] Andrews, et al. [25] Macias, et al. [26]

Clinical variation: Variation in the clinical context of wilderness medicine taught with simulation, such as multiple different patient scenarios, was evident in 10 of the published articles [15, 21-26, 28-30].

Cognitive interactivity: The use of technology-enhanced simulation to promote the cognitive engagement of learners with strategies, such as task variation, multiple repetitions, and feedback, was described in 10 articles [15, 21-26, 28-30].

Curricular integration: The incorporation of simulation intervention as an integral part (required or a formal element) of a wilderness medicine curriculum or training program was described in one article [26].

Distributed practice: Distributed practice, defined as training that is spread over a period of time, was not detailed in any of the literature.

Feedback: Description of the feedback of information on learner performance by the instructor, a peer, or a computer, either during or after the simulation activity, was evident in eight articles [15, 21-22, 25-27, 29-30].

Group practice: Technology-enhanced simulated training activities involving two or more learners was described in 10 articles [15, 21-27, 29-30].

Individualized learning: Four articles described simulation-based training that was responsive to or adapted to learner needs depending on performance [21, 24-26].

Mastery learning: None of the articles in this review clearly described a training model in which a learner must attain a clearly defined standard of performance before qualifying or advancing to the next task.

Multiple learning strategies: Eight articles described a range of different instructional strategies used to facilitate learning, such as simulated patient cases, procedural training models, intentional sequencing or task variation, feedback, and case discussions [21-26, 29-30].

Range of task difficulty: Five articles described the use of technology-enhanced simulation to provide variation in the difficulty or complexity of the tasks [15, 21, 24-26].

Repetitive practice: The opportunity for learners to experience more than one attempt at a task or performance was described in three articles [24-26].

### Outcome measures

Table 3 shows the outcome measures (modified from Kirkpatrick, 1994 and Mosley, et al. 2012 [17-18]) of this review.

**Table 3:** Outcomes Levels of Articles Reviewed Outcome levels modified from Kirkpatrick, 1994 and Mosley, et al. 2012 [17-18]

Kirkpatrick Level		Definition	Number of studies
1	Reaction to learning experience	Evidence of learners’ views on the overall learning experience, rather than any specific learning outcomes.	Saxon, et al. [21] Lockwood, et al. [22] Fielding [23] Andrews, et al. [25] Macias, et al. [26] Vohra, et al. [29] Lareau, et al. [30]
2a	Modification of attitudes and perceptions	Evidence of changes in attitudes or perceptions of learners and possible changes in perception or attitude towards the value and/or use of team approaches to caring.	Saxon, et al. [21] Lockwood, et al. [22] Mazoyer, et al. [26] Andrews, et al. [25]
2b	Acquisition of knowledge and skills	Evidence of knowledge and/or skills acquisition immediately following completion of a course/educational intervention.	Saxon, et al. [21] Lockwood, et al. [22] Heiner, et al. [28]
2c	Retention of knowledge and skills	Evidence of the retention of knowledge and/or skills over a period of time after the course/ educational intervention.	
3	Behavioral change	Evidence of transfer of learning to clinical practice.	
4a	Change in organizational practice	Evidence of changes within the organizational practice and delivery of care after the course/ educational intervention.	
4b	Benefits to patients/ clients, families and communities	Evidence of documented impacts in the health or well-being of patients/clients, families, and communities after the course/educational intervention.	

Kirkpatrick Level 1 - Reaction to the learning experience: Seven articles provided evidence of the views of the learners on the overall learning experience, rather than any specific learning outcomes [21-23, 25, 29-30].

Kirkpatrick Level 2a - Modification of attitudes and perceptions: Evidence of changes in attitudes or perceptions of learners and possible changes in perception or attitude towards the value and/or use of team approaches to caring was described in four articles [21-22, 24-25].

Kirkpatrick Level 2b - Acquisition of knowledge and skills: Three articles described evidence of knowledge and/or skills acquisition immediately following the completion of a course/educational intervention [21-22, 28].

Kirkpatrick Level 2c - Retention of knowledge and skills: No evidence of the retention of knowledge and/or skills over a period of time after the course/educational intervention was described.

Kirkpatrick Level 3 - Behavioral change: No evidence of the transfer of learning to clinical practice was described.

Kirkpatrick Level 4a - Change in organizational practice: No evidence of changes within the organizational practice and delivery of care after the course/educational intervention were described.

Kirkpatrick Level 4b - Benefits to patients/clients, families, and communities: No evidence of documented impacts in the health or well-being of patients/clients, families, or communities after the course/educational simulation was described.

## Discussion

As it was shown from the article characteristics (Table 1), there is a broad range of designs, participants learning, and clinical topics described. This trend exhibits how versatile technology-enhanced simulation education can be. Most of the simulations were conducted outside. This is likely the case to increase the fidelity of the wilderness simulations, although it is recognized that this isn’t always feasible [15, 21-27]. It is promising to see that the majority of the studies used medical students as participants, and therefore, simulation is a core part of basic training and not an optional extra post-qualification [15, 21-17, 29-30]. However, the fact that some studies used a range of training level of healthcare learners contributes that anyone can, and is, using simulation-based training [30]. From the analysis of the clinical topics described, technology-enhanced simulation education was very diverse with both clinical and non-clinical skills being developed. Likewise, it is reassuring to see the use of the technology in courses is not only prevalent but also being validated by evaluations and even developed by innovations.

In terms of the instructional design components described in the wilderness literature to date, the technology-enhanced simulation training methodologies employed are aligned closely to those considered to be effective in other aspects of healthcare (Table 2) [5, 7]. The themes of cognitive interactivity, clinical variation, feedback, group and individualized learning, and multiple learning strategies appear to be employed in most wilderness simulation-based teaching. However, interestingly, both the concepts of repetitive training (a key aspect of experiential learning) and mastery learning do not feature in the literature. It is possible to argue that this omission has been evident in hospital-based simulation training also, with the concept of deliberate practice emerging as a novel approach being a relatively recent event [31]. A lack of curricula integration at this stage may be interpreted as a reflection on the current maturing evidence base for technology-enhanced simulation as an effective modality to teach wilderness medicine.

The evaluation of the outcomes (Table 3) of wilderness courses or educational interventions that use simulation modalities is limited to that of the reaction of the learners, the modification of attitudes and perceptions, and to the acquisition of knowledge and skills [21-24, 28-30]. To summarize, the current wilderness medicine literature is descriptive and mainly focuses on how to effectively utilize technology-enhanced simulation. Evaluation of the level of satisfaction of learners with this modality is evident in the majority of articles, and in several cases, learners informed the readership with direct insights on learning wilderness medicine using simulation-based education. However, there are no current studies of behavioral change, change in organizational practice, or patient outcomes. It is worth noting that, as with other healthcare arenas, there may be an effective translation of technology enhanced learning that has improved patient outcomes but has either not been reported or there is no method of assessing and reporting this as yet. This mirrors previous reviews of the effect of hospital-based simulation education up to approximately five years ago. The value proposition of simulation education in the hospital setting has now been demonstrated, two decades after introduction and uptake as a mainstream educational modality [3-4, 10-14, 32]. Therefore, it appears technology-enhanced simulation education is behind and uptake is needed to advance the field.

This review highlights the need to strengthen the evidence base for the use of technology-enhanced simulation to teach wilderness medicine. The current literature provides a strong foundation, particularly with respect to teaching medical students [15, 21-27, 29]. Further work is needed to explore in more depth what works, for whom, and how in the context of wilderness medicine for other members of the inter-professional teams that manages patients. An exploration of the potential benefit of repetitive and mastery learning techniques and a further focus on curricular integration may align simulation-based wilderness medicine teaching with that of other healthcare disciplines. As technology evolves and human patient simulators become less expensive and more adaptable to adverse weather conditions, the opportunity to increase the utilization of human patient simulators to teach wilderness medicine should improve.

However, since simulated or standardized patients have been used to teach patient assessment and management in the wilderness setting for a decade or more, it is surprising that this review of the current literature revealed a relatively low number of articles exploring the use of technology-enhanced simulation as an educational modality in wilderness medicine [15]. This theme has been noticed before. Lareau, et al. described the use of a high fidelity human patient simulator to teach an advanced wilderness life support course and conclude that such simulators are an underused tool in wilderness medicine [30].

This finding may be explained by the perception that the cost of simulators and adverse conditions for such training devices is not worth any potential benefit of improved learning. This is suspected as human patient simulators are expensive and can cost in excess of $20,000; therefore, the value of using such devices over low-cost innovations or standard traditional techniques must be substantial and clearly publicized.

Thankfully, how to achieve cost-effective learning was a recurrent theme in many of the articles in this review and many suggested how best to make technology-enhanced simulation education as cost effective as possible. The article by Donelan in 2000 highlighted how effective experiential learning in wilderness medicine is using moulages that allow learners to develop knowledge, skills, and attitudes at a limited financial cost [15]. Likewise, Saxon, et al. described in detail how to optimally create and effectively use low fidelity wilderness simulators at low cost, including cricothyroidotomy, needle thoracocentesis, and lateral canthotomy [21]. Similarly, a low-cost solution to develop diagnostic skills to determine fractures with portable ultrasonography has been described by Heiner and McArthur and Vohra and Spano who reported a successful, novel, cost-effective, interactive part-task training tool for envenomation emergencies [28-29]. Cost savings were also highlighted as possible in terms of selection of a location for technology-enhanced simulation training sessions outdoors [22]. 

Another reason why there may be limited research on technology-enhanced simulation in wilderness training is robustness. Although the simulation industry has a long history of supplying simulators to the military where durability is a premium prerequisite, watertight, and completely weatherproof, human patient simulators are still not yet available [16].

### Limitations and strengths

This review has a number of limitations. First, the low number of articles yielded precluded any quantitative analyses. Second, the majority of the articles included are descriptive in nature as opposed to studies aiming to address specific objectives. As such, the articles are heterogeneous in nature. This review has emphasized similarities, but it must be kept in mind that descriptive articles may lack all the details of context and content to allow rigorous comparison. Third, in an attempt to provide a comprehensive overview of the evidence to date, we have kept our scope broad in terms of the instructional design characteristics inclusion criteria.

Conversely, this study has a number of strengths. The study team has expertise in both technology-enhanced simulation education theory (RM) and practicing and instructing wilderness medicine (CH). Additionally, the team benefitted greatly from librarian/informatics support to design and perform the search strategy. Also, another strength of this study is that the approach was the broad inclusion criteria with reproducible data abstraction and coding. The authors conducted all aspects of the review process independently and in duplicate, and there was a consensus so data are robustly valid.

## Conclusions

This review has answered its first two research objectives to establish what characteristics of technology-enhanced simulation have been presented in the wilderness medicine literature to date and how has the technology been used. Standardized/simulated patients, low-cost technology-enhanced solutions, and the limited use of human patient simulation has, to date, been evaluated as an effective educational modality to teach wilderness medicine. Likewise, the current provision of wilderness medicine utilizing technology-enhanced simulation has been found to be aligned with instructional design characteristics associated with effective learning.

However, further development of this field must be encouraged to answer the final research question as to whether technology-enhanced simulation can be associated with improved learner outcomes and patient outcomes. This development could be to include repetitive and mastery learning techniques. Alternatively, to increase the generalizability of conclusions of what and how technology has been used in wilderness medicine, cohorts beyond medical student could be investigated. A final suggestion is to target research towards the effectiveness of technology-enhanced learning in wilderness medicine as an educational modality that improves patient outcomes. 
